# PNP as a Metabolic and Prognostic Driver of Breast Cancer Aggressiveness: Insights from Patient Tissue and Cell Models

**DOI:** 10.32604/or.2025.070808

**Published:** 2025-12-30

**Authors:** Sarra B. Shakartalla, Iman M. Talaat, Nival Ali, Shahenaz S. Salih, Zainab M. Al Shareef, Noura Alkhayyal, Riyad Bendardaf, Sameh S. M. Soliman

**Affiliations:** 1 Research Institute for Medical and Health Sciences, University of Sharjah, Sharjah, 27272, United Arab Emirates; 2 College of Medicine, University of Sharjah, Sharjah, 27272, United Arab Emirates; 3 Faculty of Pharmacy, University of Gezira, Wadmedani, 21111, Sudan; 4 Faculty of Medicine, Alexandria University, Alexandria, 21131, Egypt; 5 College of Medical Laboratory Sciences, Sudan University of Science and Technology, Khartoum, 11111, Sudan; 6 Histopathology and Laboratory Medicine Department, University Hospital Sharjah, Sharjah, 72772, United Arab Emirates; 7 Medical Oncology Unit, University Hospital Sharjah, Sharjah, 72772, United Arab Emirates; 8 College of Pharmacy, University of Sharjah, Sharjah, 27272, United Arab Emirates

**Keywords:** Purine nucleoside phosphorylase, metastasis, hypoxanthine, breast cancer

## Abstract

**Objectives:**

Breast cancer (BC) is the leading cause of cancer-related mortality in women, largely due to metastasis. This study aims to explore the role of purine nucleoside phosphorylase (PNP), a key enzyme in purine metabolism, in the aggressiveness and metastatic behavior of BC.

**Methods:**

A comprehensive analysis was performed using *in silico* transcriptomic data (*n* = 2509 patients), immunohistochemical profiling of BC tissues (*n* = 103), and validation through western blotting in multiple BC cell lines. Gene expression and survival analyses were conducted using Tumor Immune Estimation Resource (TIMER), Gene Expression Profiling Interactive Analysis 2 (GEPIA2), and the cBioPortal for cancer genomics (cBioPortal) platforms. Correlations between PNP and key epithelial–mesenchymal transition (EMT) markers, molecular subtypes, tumor grades, and stages were examined.

**Results:**

PNP was significantly overexpressed in human epidermal growth factor receptor 2 (HER-2)-positive and triple-negative BCs compared to luminal subtypes. High PNP levels were strongly associated with advanced BC stages, high-grade tumors, EMT phenotypes, and poor overall survival. Notably, HER-2 inhibition suppressed PNP expression, while PNP gene silencing induced HER-2 upregulation, revealing a reciprocal regulatory loop. Dual inhibition of PNP and HER-2 resulted in a significant reduction in cell viability compared to HER-2 inhibition alone.

**Conclusion:**

Collectively, PNP emerges as a promising biomarker of BC aggressiveness and progression. Its reciprocal interaction with HER-2 underscores its potential as a therapeutic target. Dual targeting of PNP and HER-2 may offer a novel strategy for improving outcomes in aggressive BC subtypes.

## 1 Introduction

Breast cancer (BC) is the most prevalent malignancy among women globally [1]. It is the leading cause of mortality in females, with ~90% of deaths attributed to metastasis [2]. The unpredictable nature of metastasis highlights the importance of discovering novel, effective, specific, or generalized biomarkers that can help oncologists in customizing treatment approaches [3]. BC can be categorized into two main types: invasive and non-invasive (*in situ*). The majority of BCs are invasive, spreading beyond the ducts and glands into lymph nodes and surrounding tissues [4]. There are 21 distinct subtypes of BC, each is classified based on its histological characteristics [5]. Additionally, BC is categorized into four distinct intrinsic molecular subtypes, each differs in clinical characteristics and treatment requirements: luminal A, luminal B, human epidermal growth factor receptor-2 overexpression (HER-2+), and triple negative (TNBC) [6]. This classification relies on the status of a set of molecular markers, including the estrogen receptor (ER), progesterone receptor (PR), HER-2 (ERBB2) receptor, and mitotic index (Ki-67) [6].

A hallmark of tumorigenesis is the alteration of cellular metabolism, which is reprogrammed to support anabolic growth and survival throughout the advancement of BC [7]. Recently, we have unveiled the notable impact of hypoxanthine in promoting epithelial-mesenchymal transition (EMT) as well as enhanced migration and invasion of MCF-7 BC cells [8]. We have also demonstrated the crucial role of purine nucleoside phosphorylase (PNP) in BC cell aggressiveness and metastasis. PNP plays an important role in purine metabolism through the salvage pathway, which represents the major pathway that covers most cells’ requirements of purines [9]. Purines are vital metabolic substrates for all living organisms, offering crucial elements for DNA and RNA synthesis [10]. In addition to their role as DNA and RNA building blocks, purines supply the essential energy and cofactors that support cell survival and proliferation [10]. Consequently, purines and their derivatives are extensively involved in various biological processes, including immune responses, cell cycle, and signal transduction [10].

PNP, also referred to as purine nucleoside: orthophosphate ribosyltransferase that catalyzes the reversible transformation of purine ribosides to their corresponding bases. Specifically, it transforms inosine to hypoxanthine and guanosine to guanine [11]. PNP exists in different parts of the human body, notably in the cytosol and mitochondria. However, its activity is high in peripheral red blood cells, blood granulocytes, and lymphocytes [12]. Impaired purine metabolism is associated with the progression of cancer [13]. It has been demonstrated that patients with bronchogenic carcinoma exhibit elevated levels of PNP activity in lymphocytes [14]. Additionally, it is reported that the level of PNP is increased in tissues of the oral cavity affected by non-Hodgkin lymphomas and in prostate cancer tissues [15,16]. Furthermore, it has been established that PNP is associated with colon cancer aggressiveness [15]. However, reduced PNP activity in leukemic cells has also been documented, which is thought to result from impaired enzyme synthesis due to disruptions in the regulatory processes governing purine metabolism [17].

In this study, we investigate the direct role of PNP in EMT, as well as in the progression and prognosis of BC. Our approach combined *in silico* analysis of patient data with subsequent experimental validation on both tissue and cell levels to gain a comprehensive understanding of the correlation between PNP and BC metastasis.

## 2 Materials and Methods

### 2.1 In Silico Analysis

To identify the correlation between *PNP* gene and different EMT markers including E-cadherin (CDH1), claudin (*CLDN1*), N-cadherin (*CDH2*), vimentin (*VIM*), fibronectin (*FN1*), snail (*SNAI1*), slug (*SNAI2*), and MMP-9 (*MMP9*) and between *PNP* and BC histological biomarkers encompassing ER (*ESR1*), PR (*PRG*), HER-2 receptor (*ERBB2*), and Ki-67 (*MKI67*), tumor immune estimation resource TIMER 2.0 web platform, available at http://timer.cistrome.org was employed [18] which applies the Spearman correlation method, based on The Cancer Genome Atlas (TCGA) BC dataset (Breast cancer (BRCA), *n* = 1100). TIMER 2.0 is renowned for its robust and comprehensive data analysis capabilities, particularly in the context of gene expression profiling within various cancer types [18]. To validate our findings, we conducted an independent analysis using the gene expression profiling interactive analysis (GEPIA2) platform (http://gepia2.cancer-pku.cn/) with TCGA-BRCA data, also employing the Spearman correlation method to ensure methodological consistency. In GEPIA2, “TCGA Tumor” was selected as the dataset source, and BRCA was chosen as the cancer type. The results from both platforms were compared to assess reproducibility.

To explore the differential expression of the *PNP* gene between tumors and normal tissues, the expression analysis module of GEPIA2, version 2 web server was used, with *p-*value (*p*) cutoff = 0.01 and log_2_ fold change cutoff = 1 [19].

### 2.2 Analysis of In Vivo Data from Publicly Available Sources

To investigate the relationship between PNP and clinicopathological characteristics of BC, the publicly accessible cBioPortal for cancer genomics (cBioPortal) database (https://www.cbioportal.org/) was employed. Specifically, we used the BC dataset from METABRIC, Nature 2012, and Nat Communication 2016 [20,21]. This dataset comprises data from 2509 patients with BC, providing a substantial cohort for our analysis. The same dataset was used to identify the relationship between PNP and overall survival. The cBioportal platform employs Kaplan–Meier survival curves with the log-rank test to assess statistical significance.

### 2.3 Ex Vivo Analysis

#### 2.3.1 Clinical Samples

A total of 103 BC patients were included in the study. Among them, 69 were diagnosed with BC and surgically treated at Sharjah Breast Care Centre, University Hospital Sharjah (SBCC-UHS), while the remaining 34 received surgical treatment at Al Qassimi Hospital, Sharjah. Inclusion criteria: Female patients with histologically confirmed primary BC, who underwent mastectomy and for whom formalin-fixed, paraffin-embedded (FFPE) tumor specimens were available. Exclusion criteria: Patients who had received neoadjuvant chemotherapy or radiotherapy prior to surgery, and specimens with insufficient tumor tissue for immunohistochemical analysis.

At the hospital, formalin-fixed mastectomy specimens were sectioned, followed by tissue processing and creation of formalin-fixed paraffin-embedded (FFPE) blocks. The study was carried out in accordance with the principles of the Helsinki Declaration and was authorized by the University Hospital Sharjah Ethical and Research Committee (Ref. No.: UHS-HERC-107-14092022). Written informed consent was obtained from all patients prior to their enrollment in the study. Table 1 summarizes the demographic information and clinicopathological characteristics of the patients.

**Table 1:** Demographic and clinicopathological characteristics of BC patients

Parameters	Total no. of samples = 103	
**Age [N = 103]**	<50	51 (49.5%)
	≥50	52 (50.5%)
**Greatest tumor dimensions [N = 98]**	Mean ± SD	9.11 ± 16.39
**Tumor Diagnosis: *n* (%) [N = 103]**	Invasive carcinoma	97 (94.2%)
	Non-invasive carcinoma	6 (5.8%)
**DCIS: *n* (%) [N= 94]**	Absent	48 (51.1%)
	Present	46 (48.9%)
**Nottingham Grade: *n* (%) [N = 101]**	Grade 1	11 (10.9%)
	Grade 2	37 (36.6%)
	Grade 3	53 (52.5%)
**Molecular Subtype: *n* (%) [N = 93]**	Luminal A & Luminal B HER-2 Negative	45 (48.4%)
	Luminal B HER-2 positive & HER-2 overexpression	25 (26.8%)
	TNBC	23 (24.7%)
**TILs: *n* (%) [N = 59]**	Negative	3 (5.1%)
	Moderate	42 (71.2%)
	High	14 (23.7%)
**Positive Lymph Nodes: *n* (%) [N= 97]**	Absent	40 (41.2%)
	Present	57 (58.8%)
**Lympho-Vascular Invasion: *n* (%) [N = 88]**	Present	48 (45.5%)
	Absent	40 (45.5%)
**Stage: *n* (%) [N = 101]**	Stage 0 & 1	29 (28.7%)
	Stage 2	44 (43.6%)
	Stage 3 & 4	28 (27.7%)

**Note: BC,** Breast cancer**; DCIS,** Ductal carcinoma *in situ***; HER-2,** Human epidermal growth factor 2**; TILs,** Tissue infiltrating lymphocytes**; TNBC,** Triple negative breast cancer**; SD,** Standard deviation.

#### 2.3.2 Histopathological Examination

The histopathological examination was conducted at UHS. Briefly, FFPE BC tissues were cut into 5 μm thick slices using a sledge microtome (HM355S, Microm, Thermo Fisher Scientific, Langenselbold, Germany) and stained with hematoxylin (#6765003, Richard-Allan Scientific, Thermo Fisher Scientific, Waltham, MA, USA) and eosin (#6766007, Richard-Allan Scientific, Thermo Fisher Scientific, Waltham, MA, USA) (H&E). The 2012 WHO classification of breast tumors was used to guide histopathological typing [22]. The immunostaining of ER, PR, and HER-2 receptor, as well as the proliferation marker Ki-67, was used to identify the molecular subtyping of BC cases (Table 2).

**Table 2:** Histopathological biomarkers of BC patients

Variables		Total no. of samples = 103
**ER: *n* (%) [N = 98]**	Negative	30 (30.6%)
	Positive	68 (69.4%)
**PR: *n* (%) [N = 94]**	Negative	38 (40.4%)
	Positive	56 (59.6%)
**HER-2: *n* (%) [N = 93]**	Negative	60 (64.5%)
	Positive	24 (25.8%)
	Equivocal	9 (9.7%)
**Ki 67: *n* (%) [N = 90]**	Negative (<14%)	21 (23.3%)
	Positive (>14%)	69 (76.7%)

**Note: BC,** Breast cancer**; ER,** Estrogen receptor**; PR,** Progesterone receptor**; HER-2,** Human epidermal growth factor receptor 2.

#### 2.3.3 Immunohistochemistry

For immunostaining, FFPE BC tissues were cut into 4 μm thick slices. To melt the wax, the slides were incubated for 30 min at 60°C. Deparaffinization was then carried out by immersion twice in 100% xylene (Sigma-Aldrich, Baden-Württemberg, Germany). Following this, the slides were rehydrated for 5 min in 100% ethanol, 90% ethanol, 70% ethanol, and Milli-Q water. Afterwards, the slides were submerged in citrate buffer (pH 6) and microwaved for 15 min to achieve antigen retrieval. Immunohistochemistry (IHC) was performed using the Abcam IHC kit (#ab64264, Abcam, Cambridge, UK) according to the manufacturer’s instructions, with slight modifications. The endogenous peroxidase was blocked using the hydrogen peroxidase block (#ab64264, Abcam, Cambridge, UK) for 30 min. Following this, tissue sections were incubated with PNP antibody (# MAB6486, R&B systems, Minneapolis, MN, USA) (12 μg/mL, 1:42 dilution ratio) overnight at 4°C and subsequently incubated with biotinylated goat anti-polyvalent antibody included in the kit and without further dilution (#ab64264, Abcam, Cambridge, UK). Detection was done using diaminobenzidine substrate (DAB) (#ab64264, Abcam, Cambridge, UK), followed by counterstaining using haematoxylin and sequential dehydration with 70% ethanol, 90% ethanol, 100% ethanol, and xylene. Finally, mounting was done using the mounting agent distrene plasticiser xylene (DPX) (#GRM655, HIMEDIA, Mumbai, Maharashtra, India).

#### 2.3.4 Interpretation of Immunohistochemical Staining

PNP protein immunostaining was evaluated using an Olympus BX43 microscope equipped with an Olympus camera DP 74 (Olympus, Tokyo, Japan). The immunoreactive score (IRS) was generated by the multiplication of the staining intensity and the percentage of immuno-stained cells and designated as negative (IRS = 0), weak (IRS = 1–4), moderate (IRS = 5–7), and strong (IRS = 8–9) [23].

### 2.4 In Vitro Analysis

#### 2.4.1 Cell Cultures

MCF-7, MDA-MB-231, BT474, and SKBR3 cell lines were purchased from the American Type Culture Collection (ATCC) (Manassas, VA, USA). These cells were cultured in Dulbecco’s modified Eagle medium **(**DMEM) (#8537, Sigma-Aldrich, Germany) supplemented with 10% fetal bovine serum (#F9665, Sigma-Aldrich, Germany) and 1% penicillin/streptomycin (#P4458, Sigma-Aldrich, Germany). Additionally, human mammary epithelial cells (HME1), also procured from ATCC (Manassas, VA, USA), were grown in DMEM-F12 medium (#SLM-243-B, Sigma-Aldrich, Germany) supplemented with 10% fetal bovine serum and 1% penicillin/streptomycin. All cell cultures were maintained in a humidified incubator at 37°C with an atmosphere containing 5% CO_2_. All cell lines were verified to be Mycoplasma-free and authenticated using short tandem repeat (STR) profile analysis before experiments.

#### 2.4.2 PNP Gene Silencing

**Figure A1:**
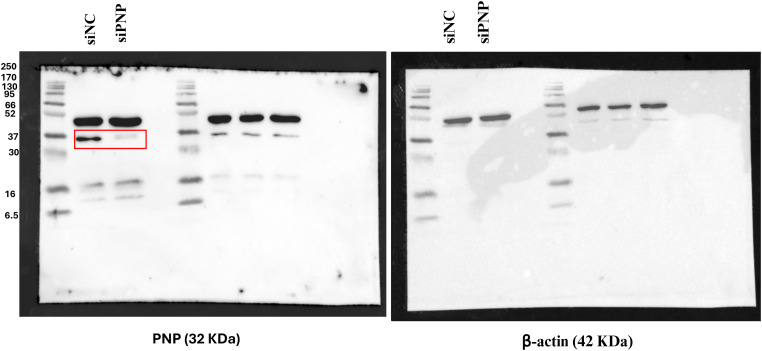
Efficiency of PNP knockdown. The figure shows the uncropped western blot of PNP and β-actin protein bands in SKBR3 cells upon Knockdown of PNP. siNC; negative control

SKBR3 cells were plated in 6-well plates at a density of 2 × 10^5^ cells per well using antibiotic-free culture medium. On the following day, transfection was performed using Lipofectamine™ RNAiMAX reagent (#13778150, Thermo Fisher Scientific, Waltham, MA, USA) in accordance with the manufacturer’s protocol. Cells were transfected with 50 nM Silencer™ Select siRNA targeting the PNP gene (#4390824, Thermo Fisher Scientific, Waltham, MA, USA). Silencer™ Select negative control siRNA (#4390844, Thermo Fisher Scientific, Waltham, MA, USA) was utilized as a negative control. After 72 h of transfection, the cells were collected for subsequent analysis. Knockdown efficiency was confirmed by a marked reduction in PNP protein band intensity in siRNA-treated cells compared to scrambled controls (Fig. A1).

#### 2.4.3 Drug Treatment

For western blotting, SKBR3 cells were seeded in 6-well plates at a density of 25 × 10^4^ cells per well. The next day, the cells were treated with 10 μM Forodesine (BCX-1777) (#HY-16210, MedChemexpress, Monmouth Junction, NJ, USA) or 10 μg/mL trastuzumab (Herceptin^®^, Roche, Basel, Switzerland). Vehicle-treated cells were used as a control. After 48 h, the cells were collected for further analysis.

For the cell viability assay, SKBR3 cells were seeded in 24-well plates at a density of 5 × 10^4^ cells per well. The next day, the cells were treated with 50 nM of siRNA targeting the PNP gene for 48 h. Subsequently, trastuzumab was added to the transfected cells at a final concentration of 10 μg/mL. Control groups included cells treated with 10 μM BCX-1777 alone, 10 μg/mL trastuzumab alone, or a combination of BCX-1777 and trastuzumab. After 48 h, the cell viability was assessed using the MTT assay.

#### 2.4.4 MTT Assay

MTT [3-(4,5-dimethylthiazol-2-yl)-2,5-diphenyltetrazolium bromide] assay was used to assess the SKBR3 cells viability. 200 μL of 1:10 MTT (#M2128, Sigma-Aldrich, St. Louis, MO, USA) solution in serum-free media was added to each well in a 24-well plate and incubated for 2 h at 37°C in the dark. Following incubation, the resulting formazan crystals were dissolved by adding 400 μL of dimethyl sulfoxide (DMSO) (#D8418, Sigma-Aldrich, St. Louis, MO, USA) to each well. The plate was shaken in a microplate shaker (BT1502, Benchmark Scientific, Sayreville, NJ, USA) for 10 min. 50 μL of the mixture was added to a 96-well plate containing 150 μL DMSO. The absorbance was measured at 570 nm using a microplate reader (Synergy H1, Biotek instruments, Winooski, VT, USA), and the readings were multiplied by 4 (dilution factor). Cell viability was expressed as a percentage relative to the untreated control group.

#### 2.4.5 Western Blot

MCF-7, MDA-MB-231, BT474, HME1, and SKBR3 cell lines were lysed using RIPA buffer (25 mM Tris/HCl pH 7.6, 150 mM NaCl, 1% NP-40, 1% sodium deoxycholate, 0.1% SDS). The protein levels in each lysate were determined with the Pierce BCA protein assay kit (#23227, ThermoFisher Scientific, Waltham, MA, USA), as per the provided guidelines. For electrophoresis, ~20–30 μg of total protein from each sample was loaded onto an SDS-PAGE gel. Following electrophoresis, the proteins were transferred to a nitrocellulose membrane (#10600004, Amersham, GE Healthcare Life Science, Freiburg, Germany), which was subsequently blocked with 5% bovine serum albumin (#A2153, Sigma-Aldrich, St. Louis, MO, USA). The membrane was then incubated with primary antibodies against PNP (dilution 1:1000) (#MAB6486, R&D Systems, Minneapolis, MN, USA), HER-2 (dilution 1:1000) (#AB214275, Abcam, Cambridge, UK), and β-actin (dilution 1:2000) (#4970, Cell Signaling Technology, Danvers, MA, USA) at 4°C overnight. Afterwards, the membranes were incubated with either anti-mouse (#7076, Cell Signaling Technology, Danvers, MA, USA) or anti-rabbit (#7074S, Cell Signaling Technology, Danvers, MA, USA) Immunoglobulin G Horseradish Peroxidase (IgG HRP)-conjugated secondary antibodies (dilution 1:1000) for 1 h at ambient temperature. Detection of the proteins was performed using the Clarity Western ECL Substrate detection system (#170-5060, Bio-Rad, Hercules, CA, USA). The experiment was done using 3 independent replicates.

### 2.5 Statistical Analyses

For immunohistochemistry, IRS were calculated by multiplying staining intensity by the percentage of positive cells, yielding a score from 0 to 9. The samples were designated as negative (IRS = 0), weak (IRS = 1–4), moderate (IRS = 5–7), and strong (IRS = 8–9) [23]. Cases with negative and weak scores of PNP were grouped as “Negative”, while moderate and strong scores were grouped as “Positive”. Normality was checked for age and the greatest dimensions using Kolmogorov-Smirnov and Q-Q plots; they were found to be not normally distributed. The Mann-Whitney U test was used to analyse differences in age and the greatest dimensions in relation to PNP levels. Pearson Chi Square and Fisher’s Exact test were used to analyze the association between PNP expression levels with the clinicopathological parameters and other biomarkers. All tests were two-tailed, and the significance level was set at *p*-value < 0.05. Data were analyzed using Statistical Package for Social Sciences (SPSS) (IBM version 23 for Windows, Armonk, NY, USA).

Kaplan–Meier survival analyses were performed using the cBioPortal platform, which applies the log-rank test by default. As the analyses were generated directly from this platform, additional verification of the proportional hazards’ assumption (e.g., Schoenfeld residuals) or alternative tests (e.g., Rényi test) could not be performed.

For *in vitro* experiments, statistical analyses were performed using GraphPad Prism (version 9.4.1, San Diego, CA, USA). Data are presented as mean ± standard deviation (SD). For comparisons between two independent groups, Student’s *t*-test was used. For comparisons among more than two groups, one-way analysis of variance (ANOVA) was performed, followed by Dunnett’s multiple comparisons test. A *p*-value < 0.05 was considered statistically significant.

## 3 Results

### 3.1 In Silico Analysis Showed a Significant Correlation between PNP and EMT, Hormonal and Proliferation Markers

**Figure 1:**
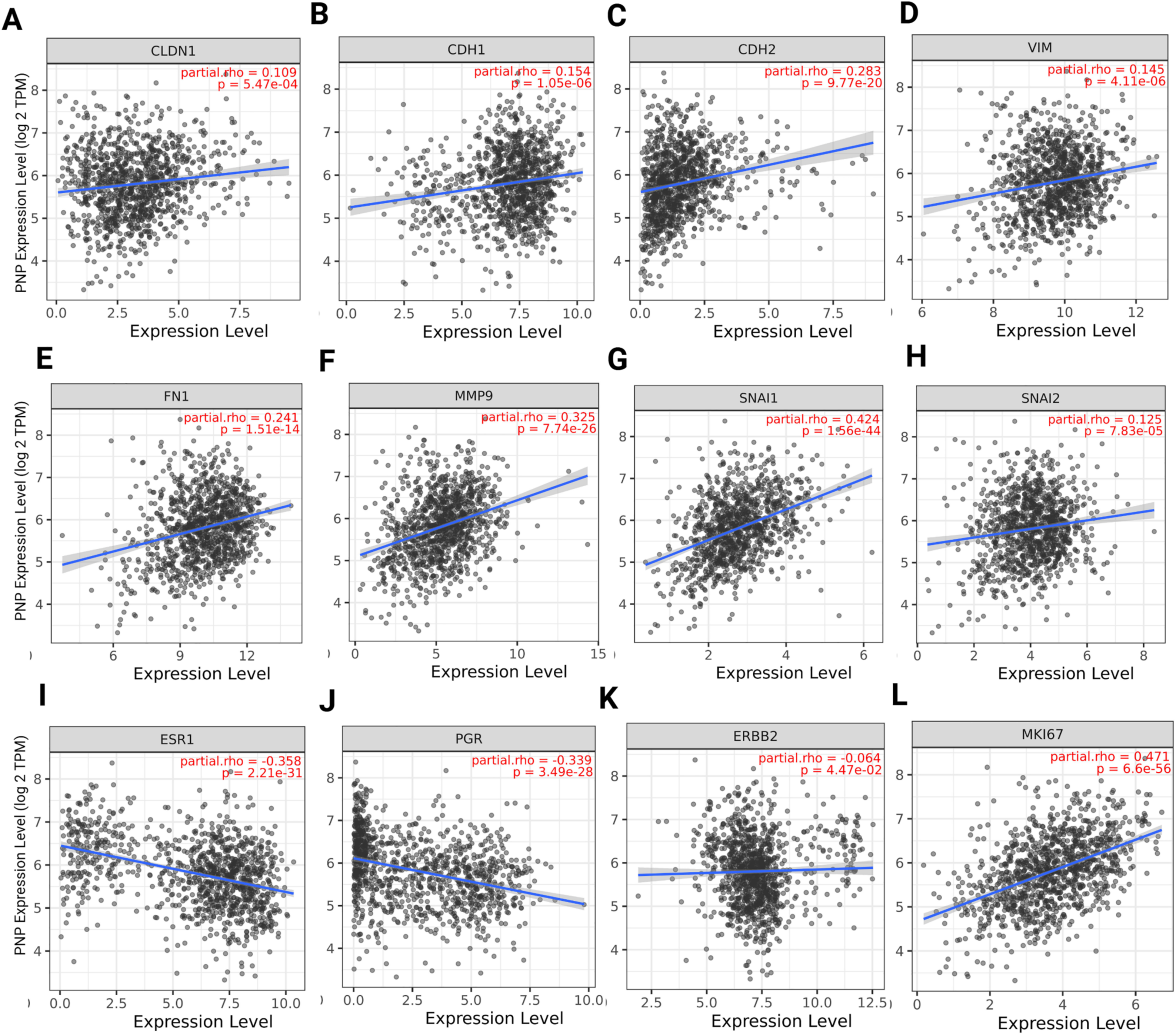
Spearman’s rank correlation between PNP and EMT, hormonal, and proliferation markers. (**A**–**H**) EMT markers, including (**A**) Claudin, (**B**) E-cadherin, (**C**) N-cadherin, (**D**) Vimentin, (**E**) Fibronectin, (**F**) MMP-9, (**G**) Snail, and (**H**) Slug. (**I–K**) Hormonal markers including (**I**) estrogen receptor, (**J**) progesterone receptor, and (**K**) HER-2. (**L**) Proliferation marker Ki-67. The figure was obtained using the TIMER 2 web tool. rho denotes Spearman’s rank correlation coefficient

For epithelial cancer cells to invade the surrounding stroma and metastasize, they undergo a phenotypic transition to a mesenchymal state, which enhances their motility [24]. In our previous study, we proved that hypoxanthine, which is a product of PNP enzyme, induces EMT in BC cells [8]. We have also demonstrated the importance of PNP in BC cell metastasis through chemical inhibition and gene knockdown. This finding encouraged us to investigate the link between PNP and EMT markers and to ascertain whether PNP is correlated with the invasive and aggressive nature of BC. Using TIMER 2.0, we have found a significant positive correlation between *PNP* and epithelial markers, including claudin (*CLDN1*) (*p* = 5.47e−04) and E-cadherin (CDH1) (*p* = 1.05e−06) (Fig. 1A,B). Similarly, a significant (*p* < 0.00001) positive correlation between *PNP* and mesenchymal markers, including N-cadherin (*CDH2*), vimentin (*VIM*), fibronectin (*FN1*), snail (*SNAI1*), slug (*SNAI2*), and MMP-9 (*MMP9*) was observed (Fig. 1C–H). These results suggest that *PNP* expression is correlated with EMT phenotype progression in BC.

**Figure A2:**
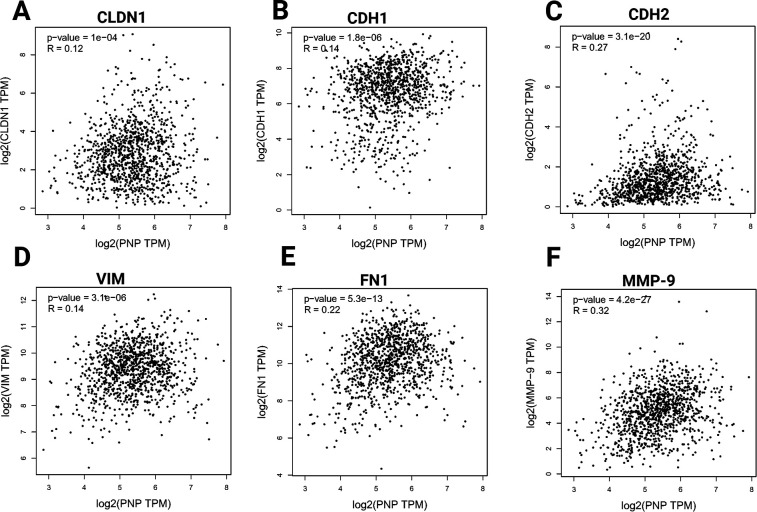

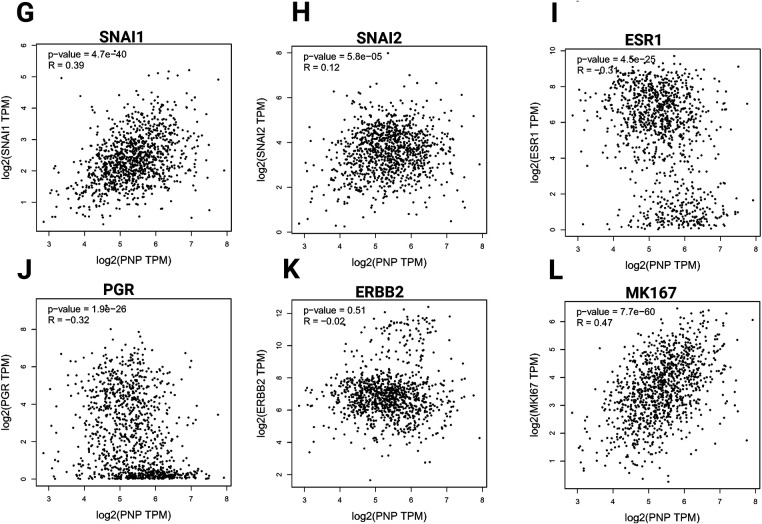
Scatter plots showing correlations between PNP expression and EMT markers, hormone receptors, and proliferation markers in BRCA (TCGA-BRCA dataset) as analyzed using GEPIA2. (**A–H**) EMT markers, including (**A**) Claudin, (**B**) E-cadherin, (**C**) N-cadherin, (**D**) Vimentin, (**E**) Fibronectin, (**F**) MMP-9, (**G**) Snail, and (**H**) Slug. (**I–K**) Hormonal markers including (**I**) estrogen receptor, (**J**) progesterone receptor, and (**K**) HER-2. (**L**) Proliferation marker Ki-67

The relation between *PNP* and histological biomarkers of BC was investigated to identify the relationship between *PNP* expression and the status of hormone receptor, HER-2, and proliferation index Ki-67. We have found that *PNP* expression exhibits significant negative correlation with hormone receptors *ESR1* (*p* = 2.2e−31) and *PGR* (*p* = 3.49e−28) (Fig. 1I,J), suggesting that higher levels of *PNP* are associated with lower expression of ER and PR. BCs that are ER and PR-negative are typically more aggressive and have fewer treatment options compared to hormone receptor-positive cancers [25]. *PNP* has a slightly negative correlation with HER-2 (*ERBB2*) (Fig. 1K). HER-2 overexpression is associated with a more aggressive form of BC that can be targeted with specific therapies [25]. The negative correlation with these receptors indicates that BCs with higher *PNP* levels might be less likely to respond to hormone therapies and anti-HER-2 treatment, potentially classifying them as TNBC, which is more challenging in the treatment [25]. On the other hand, *PNP* expression is positively correlated with Ki-67 proliferation markers (*p* = 6.6e−56) (Fig. 1L), indicating that higher *PNP* levels are associated with increased cell proliferation. This suggests that BCs with higher *PNP* expression are more aggressive due to their higher proliferation rates. Independent validation using GEPIA2 (Spearman correlation, TCGA-BRCA) produced consistent results, which are presented in Fig. A2.

### 3.2 In Silico Analysis Showed a Significant Increase in the Expression of PNP in Cancer Patients Compared to Normal Individuals

**Figure 2:**
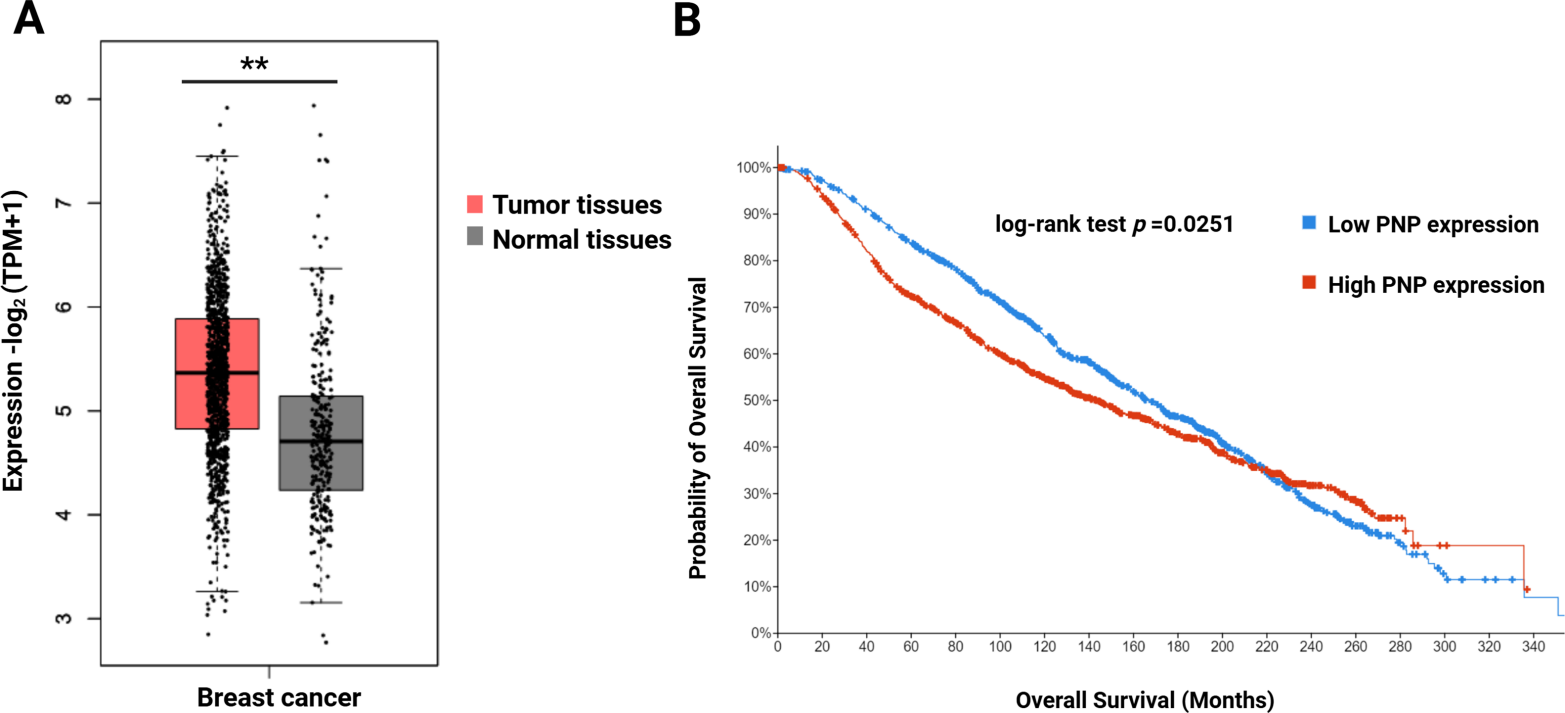
Difference in the expression level of PNP between normal individuals and survival of BC patients. (**A**) Gene expression analysis of PNP in normal (*n* = 291) and BC tissues (*n* = 1085) using the GEPIA2 web tool. (**B**) Kaplan–Meier Overall survival (OS) of BC patients using the cBioportal platform. ** reveals that *p*-value < 0.01. Note: Kaplan–Meier survival analyses were generated using the cBioPortal platform, which applies the log-rank test by default; alternative tests (e.g., Rényi) or proportional hazards verification (e.g., Schoenfeld residuals) are not available within this platform

To investigate whether there is a difference in *PNP* expression between normal and BC patients, the GEPIA2 platform was employed. A significant (*p* < 0.01) increase in the *PNP* expression in BC patients compared to healthy individuals was observed (Fig. 2A). This observation indicates that *PNP* serves as a predictive marker of BC.

### 3.3 High PNP Expression Is Associated with Poor Survival of BC Patients

To investigate the impact of PNP expression on prognosis and survival outcomes in BC patients, a cBioPortal analysis was performed. High *PNP* expression was found to be significantly (*p* = 0.0251) correlated with unfavourable survival (Fig. 2B). The median overall survival (OS) time in the group with high *PNP* expression was 168.2 months, while the median in the group expressing low *PNP* was 143.6 months.

### 3.4 Analysis of Publicly Accessible Transcriptomics Data of BC Patients Reveals a Significant Correlation between PNP and TNBC/HER-2 Positive BC

The luminal A molecular subtype is characterized by positive ER and/or PR expression and negative for HER-2, often coupled with a low histological grade [6]. This subtype is associated with the most favourable prognosis, as these cells tend to grow more slowly [6]. For luminal A cancers, the cell proliferation marker Ki-67 is typically less than 14% [6]. Luminal A cancers have the lowest likelihood of metastasis. When metastasis occurs, bones are the most commonly affected sites, yet the prognosis remains relatively good [6]. In addition, the luminal B subtype is characterized by a high histological grade and elevated levels of cell proliferation [6], resulting in a poorer prognosis compared to the luminal A subtype [6]. Within the luminal B HER-2 negative group, tumors are positive for ER and/or PR, negative for HER2, and have a Ki-67 level greater than 14%. Conversely, the luminal B HER-2 positive group exhibits ER and/or PR positivity, HER-2 positivity, and a Ki-67 level above 14% [6]. Moreover, the HER-2 overexpression subtype is marked by high levels of HER-2 oncoprotein and a lack of hormone receptors [6]. It typically presents with an intermediate histological grade [6]. The TNBC is defined by the absence of hormonal receptors and HER-2 expression [6] and characterized by a high histological grade and a high rate of mitosis. TNBC is accountable for a significant number of deaths due to its aggressive nature [26].

**Figure 3:**
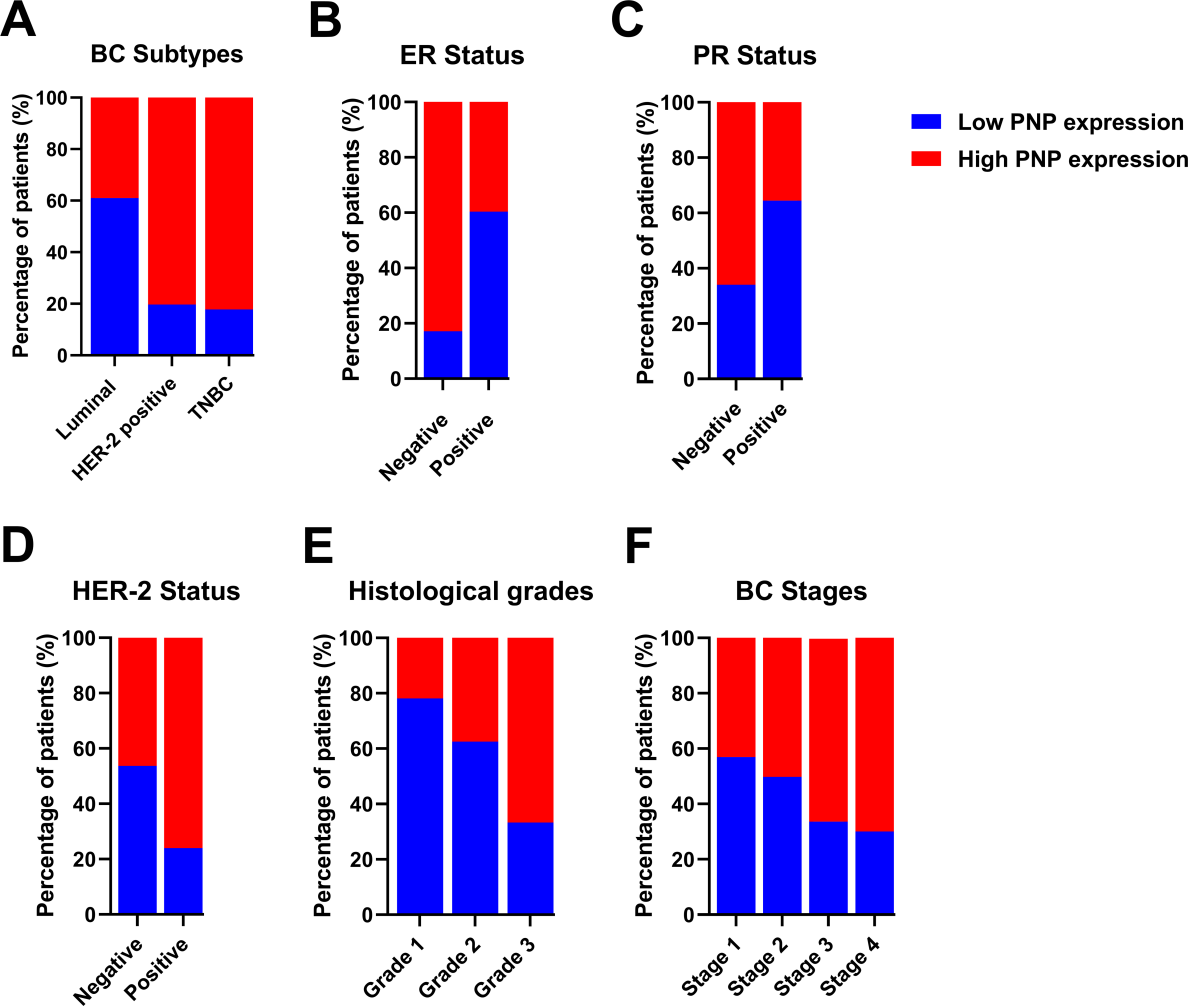
Analysis of transcriptomics data of 2509 invasive BC patients in relation to *PNP* expression and BC subtypes, stages, and grades. The association between *PNP* mRNA expression and (**A**) BC subtypes Luminal: luminal A (ER+/PR+, HER2−) and luminal B (ER+/PR+, HER2−), and HER−2 positive: luminal B (ER+/PR+, HER2+) and HER2 overexpression (ER−/PR−, HER2+) and TNBC (ER−/PR−, HER2−), (**B**) ER status, (**C**) PR status, (**D**) HER-2 status, (**E**) Histologic grades, and (**F**) BC stages. Bar charts of patients’ percentages according to high PNP expression and low PNP expression groups

Transcriptomics data of 2509 patients with invasive breast carcinoma were re-analyzed by dividing the cohort according to PNP expression into patients with high PNP expression and others with low PNP expression according to the mRNA level of *PNP*. Analysis revealed a significant association between *PNP* expression and BC subtypes (*p* < 10^−10^) (Fig. 3A). Interestingly, 82.2% of BC patients with the TNBC subtype possess high *PNP* expression levels. Similarly, a notable 80.3% with HER-2 overexpression showed high levels of *PNP* (Fig. 3A). On the other hand, a smaller fraction, 39% of patients with luminal subtype encompassing patients with luminal A and luminal B-HER-2 negative have high levels of *PNP* (Fig. 3A).

The expression of hormonal receptors in BC, specifically ER and PR, showed a significant association with *PNP* expression (*p* < 10^−10^). A large proportion of patients who were negative for ER (82.91%) exhibited high expression of PNP (Fig. 3B). Conversely, most patients who were positive for ER (60.36%) did not express *PNP* (Fig. 3B). Similarly, the majority of patients who are PR-negative (65.96%) showed positive expression of *PNP*, while 64.42% of patients who are PR-positive showed lower *PNP* expression (Fig. 3C). These results are in line with the findings obtained using TIMER-2. However, a significant portion of patients who tested positive for HER-2 (76.11%) also showed positive staining for *PNP*, a finding that is contradictory to the results reported by TIMER-2 (Fig. 3D). These results indicate that *PNP* is associated with poor prognosis and aggressiveness of BC.

### 3.5 PNP Is Significantly Correlated with Higher Histological Grades and Stages of BC

BC can be categorized based on nuclear polymorphism, tubule formation, and mitotic count into distinct histologic grades to grade 1 (low grade, well-differentiated), grade 2 (intermediate grade, moderately differentiated), and grade 3 (high grade, poorly differentiated) [5]. Cancers of a higher grade are more aggressive, exhibiting an increased tendency for recurrence and more rapid progression [5]. Transcriptomics analysis revealed a significant (*p* < 10^−10^) positive correlation with BC histological grades (Fig. 3E). The results showed that 21.89% of patients with grade 1, 37.48% of grade 2 patients, and 66.7% of grade 3 patients are highly expressing *PNP*, confirming the association between *PNP* and BC aggressiveness.

According to the Tumor–Node–Metastasis (TNM)-staging system, BC can be classified into 4 stages from 1–4 based on tumor size, regional lymph node involvement, and distant metastasis [27]. Metastatic BC is considered the last stage of BC (stage IV) [27]. *PNP* is found to be significantly (*p* = 2.652e−4) associated with cancer stages. With the advancement of BC stages, *PNP* expression is elevated; 43.11% of patients in stage I, 50.18% in stage II, 66.1% in stage III, and 70% in stage IV exhibited high levels of *PNP* expression (Fig. 3F), positivity confirming its correlation with the BC’s invasiveness and metastatic potential.

### 3.6 Ex Vivo Analysis

#### 3.6.1 Clinicopathological Characteristics of BC Patients

In a cohort of 103 BC patients, the age distribution and histological types of BC were as follows: Among the 103 patients, 51 (49.5%) were younger than 50 years old, while the remaining patients were 50 years old or older (Table 1). The majority of patients, specifically 97 (94.2%) of them, were diagnosed with invasive carcinoma, including invasive ductal carcinoma (IDC), invasive lobular carcinoma (ILC), and mixed histological types of BC, while only 6 (5.8%) of the patients were diagnosed with non-invasive carcinoma, including ductal carcinoma *in situ* (DCIS). Additionally, the classification of patients according to their molecular subtype is as follows: Luminal A and luminal B (HER-2 Negative) encompass 45 (48.4%), Luminal B (HER-2 Positive) and HER-2 enriched constitute 25 patients (26.8%), and triple-negative is represented by 23 patients (24.7%) (Table 1). The ER status was found to be negative in 30 patients (30.6%) and positive in 68 patients (69.4%) (Table 2). The PR status was negative in 38 patients (40.4%) and positive in 56 patients (59.6%). HER-2 positivity was observed in 24 patients (25.8%), while HER-2 negativity was observed in 60 patients (64.5%). The HER-2 status was equivocal in 9 patients (9.7%). The proliferation marker Ki-67 was positive in 69 patients (76.7%) and negative in 21 patients (23.3%) (Table 2).

#### 3.6.2 PNP Is Highly Expressed in BC Tissues

**Figure 4:**
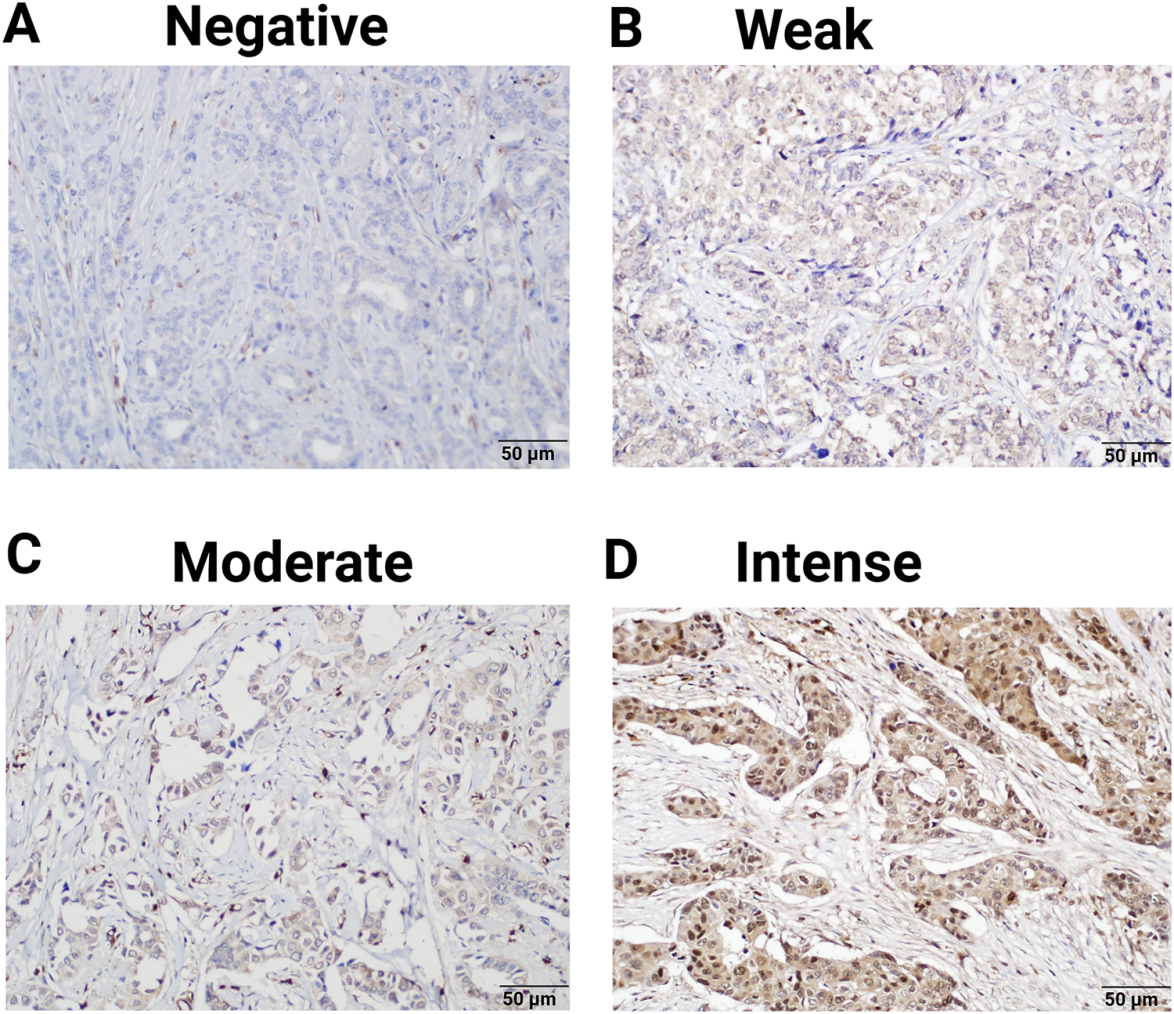
Expression of PNP in clinical breast tumors. 103 Samples were analyzed by immunohistochemistry for the expression of PNP. Samples were scored using the immunoreactive score (IRS) and designated as (**A**) negative (IRS = 0), (**B**) weak (IRS = 1–4), (**C**) moderate (IRS = 5–7), and (**D**) intense (IRS = 8–9). Magnification used is 20×

Immunohistochemical staining demonstrated that the expression of PNP was absent in 4 patients (3.9%), weak in 27 patients (26.2%), and moderately positive in 36 patients (35%). Notably, 36 patients (35%) exhibited strong positivity for PNP expression (Table 3 and Fig. 4). The cases with negative and weak scores for PNP were grouped as “Negative”, while moderate and strong scores were grouped as “Positive”. Most patients, 72 (69.9%), were positive for PNP, while 31 (30.1%) were negative (Table 3).

**Table 3:** PNP expression status in BC patients

Variables		Total no. of samples = 103 N (%)
**PNP status levels**	Negative	4 (3.9%)
	Weak	27 (26.2%)
	Moderate	36 (35.0%)
	Strong	36 (35.0%)
**PNP Status**	Negative	31 (30.1%)
	Positive	72 (69.9%)

Note: PNP, Purine nucleoside phosphorylase; BC, Breast cancer.

#### 3.6.3 PNP Expression Is Significantly Associated with BC Molecular Subtypes

**Figure 5:**
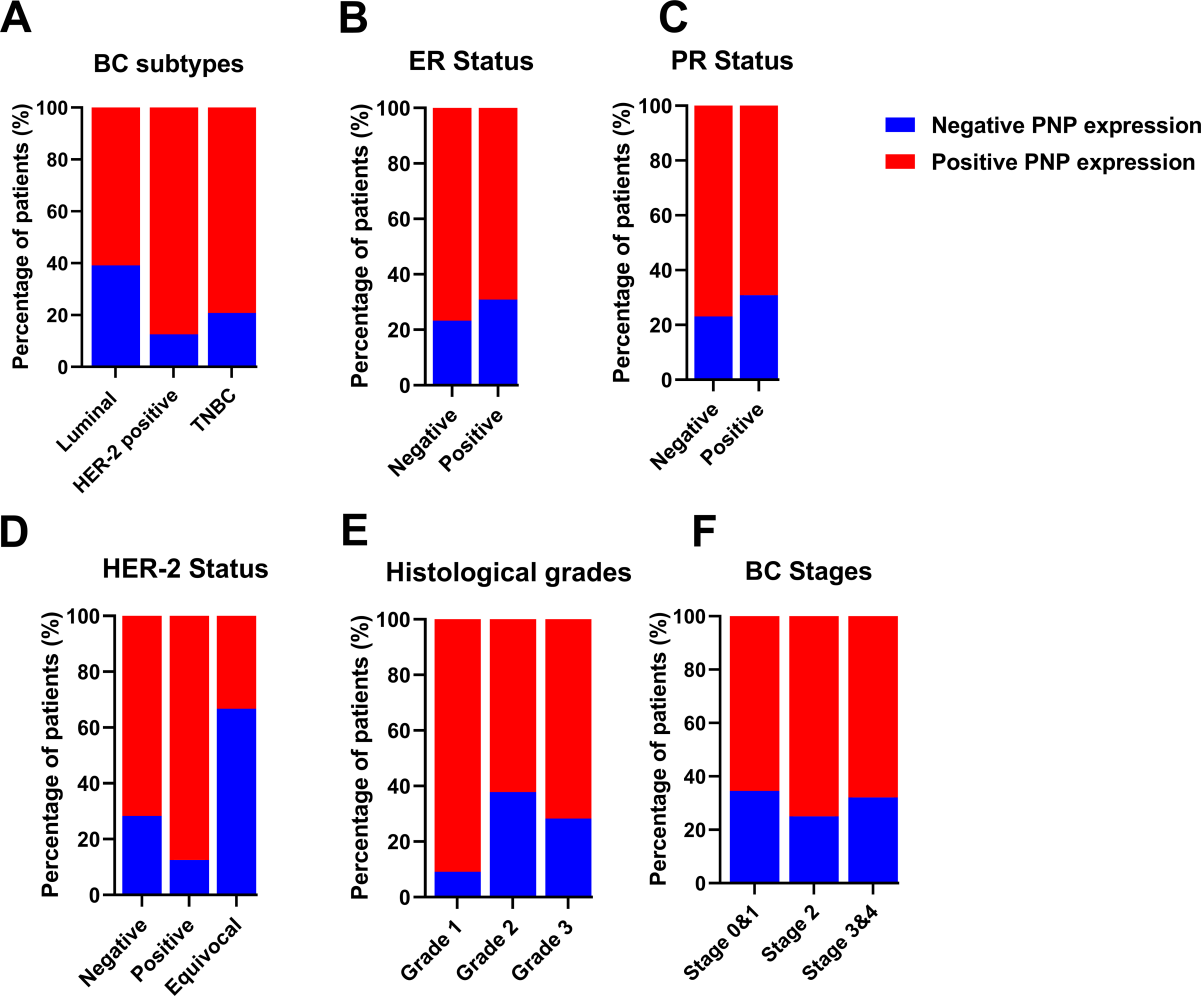
Analysis of invasive breast cancer patients’ tissues based on protein expression of PNP in relation to BC subtypes, stages, and grades. The association between PNP protein expression and (**A**) breast cancer subtypes Luminal: luminal A (ER+/PR+, HER-2−) and luminal B (ER+/PR+, HER-2−), and HER−2 positive: luminal B (ER+/PR+, HER−2+) and HER−2 overexpression (ER−/PR−, HER−2+) and triple negative (ER−/PR−, HER-2−), (**B**) ER status, (**C**) PR status, (**D**) HER−2 status, (**E**) Histologic grades, and (**F**) BC stages. Bar charts of patients’ percentages according to high PNP expression and low PNP expression groups

Our results showed that PNP is significantly (*p* < 0.05) associated with the molecular subtype of BC (Table 4 and Fig. 5A). 60.9% of patients with luminal A and HER-2 negative-luminal B expressed positive PNP. 21 (87.5%) with HER-2 overexpression and luminal B-HER-2 positive, showed high PNP positivity (Table 4). It was observed that 79.2% of patients with TNBC were positive for PNP expression. 76.7% of ER-negative patients were positive for PNP, and 76.9% of PR-negative patients were PNP positive (Table 5 and Fig. 5B,C).

**Table 4:** The association between PNP expression levels in BC patients and the clinicopathological characteristics

Variables		PNP expression levels		*p*-value*
		Negative (*n* = 31)	Positive (*n* = 72)	
**Age: Mean ± SD**		52.13 ± 16.63	50.68 ± 13.64	0.6449
**Greatest Dimensions: Mean ± SD**		7.88 ± 20.00	10.00 ± 14.60	0.5549
Diagnosis: *n* (%)	Invasive carcinoma	27 (28.1%)	69 (71.9%)	0.3299
	Non-invasive carcinoma	3 (50.0%)	3 (50.0%)	
**Molecular Subtype: *n* (%)**	Luminal A & Luminal B HER-2 negative	18 (39.1%)	28 (60.9%)	0.0420*
	Luminal B HER-2 Positive & HER-2 overexpression	3 (12.5%)	21 (87.5%)	
	TNBC	5 (20.8%)	19 (79.2%)	
**Nottingham Grading: *n* (%)**	Grade 1	1 (9.1%)	10 (90.9%)	0.1772
	Grade 2	14 (37.8%)	23 (62.2%)	
	Grade 3	15 (28.3%)	38 (71.7%)	
**TILs: *n* (%) [*n* = 58]**	Negative	0 (0.0%)	3 (100.0%)	0.2880
	Moderate	8 (19.5%)	33 (8.5%)	
	High	5 (35.7%)	9 (64.3%)	
**Positive Lymph Node: *n* (%)**	Absent	13 (32.5%)	27 (67.5%)	0.6494
	Present	15 (26.3%)	42 (73.7%)	
**Lympho-Vascular Invasion: *n* (%)**	Absent	13 (27.7%)	34 (72.3%)	0.4868
	Present	14 (35.0%)	26 (64.0%)	
**Stage Status: *n* (%)**	Stage 0&1	10 (34.5%)	19 (65.5%)	0.6495
	Stage 2	11 (25.0%)	33 (75.0%)	
	Stage 3 & 4	9 (32.1%)	19 (67.9%)	

Note: *Results are considered significant when *p*-value < 0.05; PNP, Purine nucleoside phosphorylase; BC, Breast cancer; SD, Standard deviation; HER-2, Human epidermal growth factor receptor; TNBC, Triple negative breast cancer; TILs, Tissue infiltrating lymphocytes.

**Table 5:** The association between PNP expression levels in BC patients and BC biomarkers

Variables		PNP Expression levels		*p*-value*
		Negative (*n* = 31)	Positive (*n* = 72)	
**ER Status**	Negative	7 (23.3%)	23 (76.7%)	0.4798
	Positive	21 (30.9%)	47 (69.1%)	
**PR Status**	Negative	9 (23.1%)	30 (76.9%)	0.4860
	Positive	17 (30.9%)	38 (69.1%)	
**HER-2 Status**	Negative	17 (28.3%)	43 (71.7%)	0.0085*
	Positive	3 (12.5%)	21(87.5%)	
	Equivocal	6 (66.7%)	3 (33.3%)	
**Ki-67 Status**	Positive (>14)	5 (23.8%)	16 (76.2%)	>0.9999
	Negative (<14)	19 (27.5%)	50 (72.5%)	

Note: *Results are considered significant when *p*-value < 0.05; PNP, purine nucleoside phosphorylase; BC, Breast cancer; ER, Estrogen receptor; PR, Progesterone receptor; HER-2, Human epidermal growth factor receptor-2.

The association between HER-2 and PNP was investigated. We found that PNP is significantly associated with HER-2 (*p* < 0.01) (Table 5 and Fig. 5D). 87.5% of HER-2 positive patients were PNP positive. The varying levels of PNP expression across the patients in the aforementioned results can indicate a correlation between PNP expression and the tumor’s behavior or aggressiveness. These results are consistent with the results obtained from the analysis of publicly available data, confirming the association between PNP and BC aggressiveness.

However, the results didn’t show a significant difference between patients with low or high proliferation index (Ki67) (Table 5). In addition, there was no significant difference between PNP level and different histological grades or stages in our cohort (Table 5 and Fig. 5E,F).

#### 3.6.4 PNP Protein Expression Level across Different Cell Lines Is Significantly Correlated with BC Aggressiveness and Metastasis

**Figure 6:**
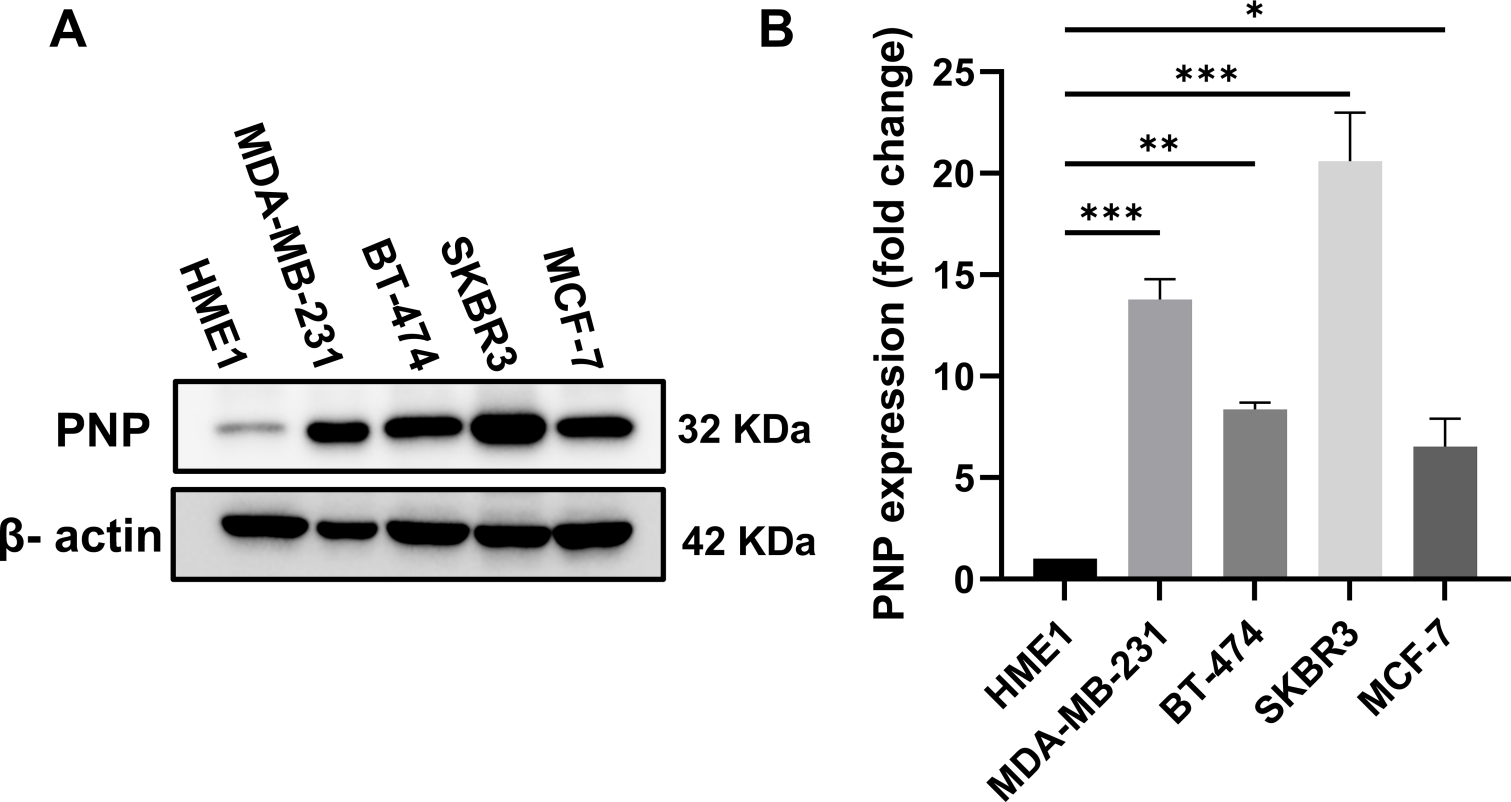
Difference in PNP protein expression in BC cell lines compared to normal mammary cells. (**A**) Western blot analysis for PNP expression among different cell lines. (**B**) Fold change in PNP protein expression between different BC cell lines relative to normal mammary epithelial cells (HME1). Data shown are representative blots from three independent biological replicates (n = 3). Quantification represents mean ± SD. The data were analyzed using one-way ANOVA. *p*-value < 0.05 was considered significant. * reveals that *p*-value < 0.05, ** reveals that *p*-value < 0.01, *** reveals that *p*-value < 0.001. Original blots are presented in Fig. A3

For further validation, a Western blot analysis was performed to measure the expression of PNP in BC cells relative to normal mammary cells. BC cell lines with distinct genetic backgrounds were used in this study (Table 6). The results showed a substantial increase in PNP protein levels. Compared to normal cells (HME1), MDA-MB-231 exhibited a 14-fold increase (*p* < 0.001), BT474 demonstrated an 8-fold increase (*p* < 0.01), SKBR3 showed a 22-fold increase (*p* < 0.001), and MCF-7 cells displayed a 5-fold increase (*p* < 0.05) (Fig. 6). Our results indicate that PNP expression is high in BC cells compared to normal mammary cells. SKBR3 and MDA-MB-231 cells exhibited the highest increase in PNP, confirming the association between PNP and BC aggressiveness and metastatic capabilities.

**Table 6:** Hormonal receptor and HER-2 status among different BC cell lines

Molecular subtype	Cell line	ER Status	PR Status	HER-2 Status
Luminal A	MCF-7	**+**	**+**	**−**
Luminal B	BT-474	**+**	**+**	**+**
HER-2 overexpression	SKBR3	**−**	**−**	**+**
TNBC	MDA-MB-231	**−**	**−**	**−**

Note: **ER**, Estrogen receptor; **PR**, Progesterone receptor; **HER-2**, Human epidermal growth factor receptor-2; **TNBC**, Triple negative breast cancer; “**+**”, positive expression; “**−**”, negative expression.

### 3.7 HER-2 Inhibition Significantly Reduced the PNP Protein Level

**Figure 7:**
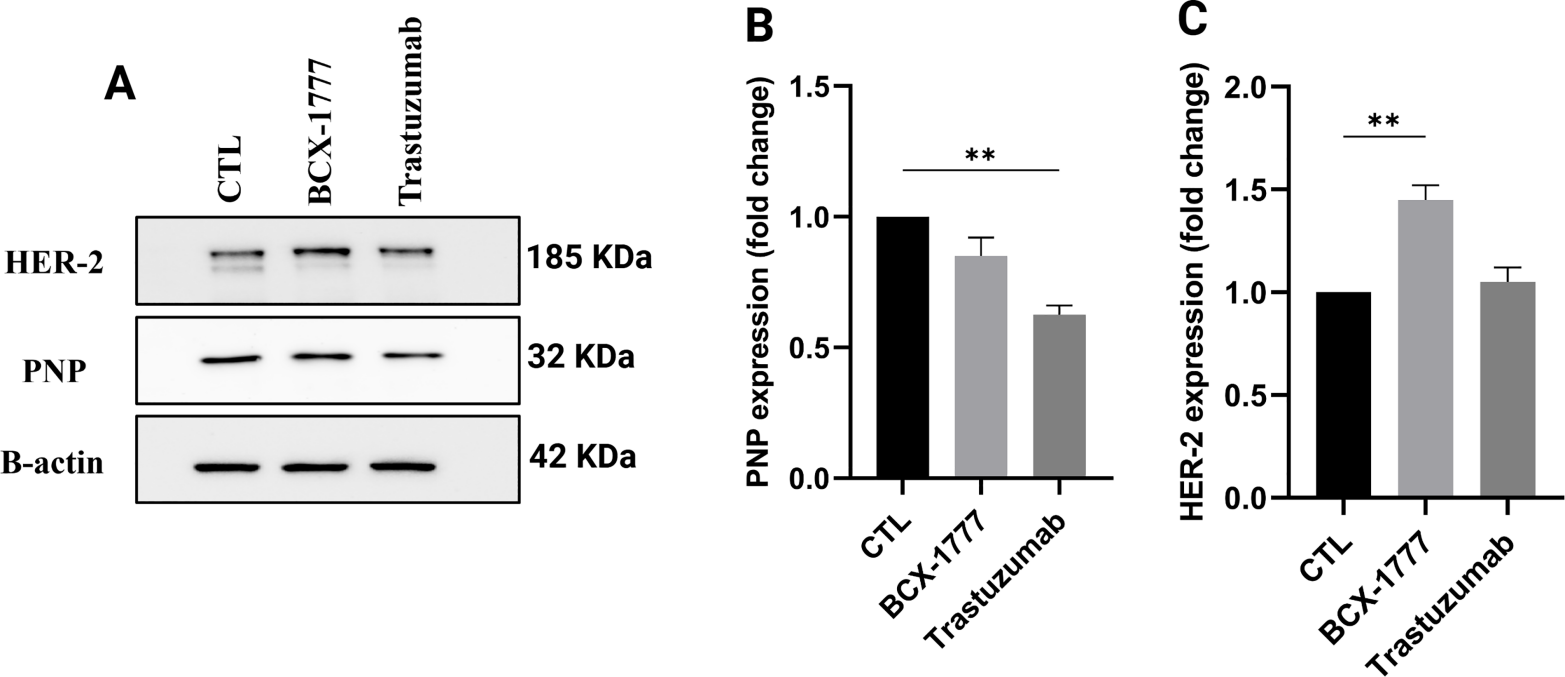

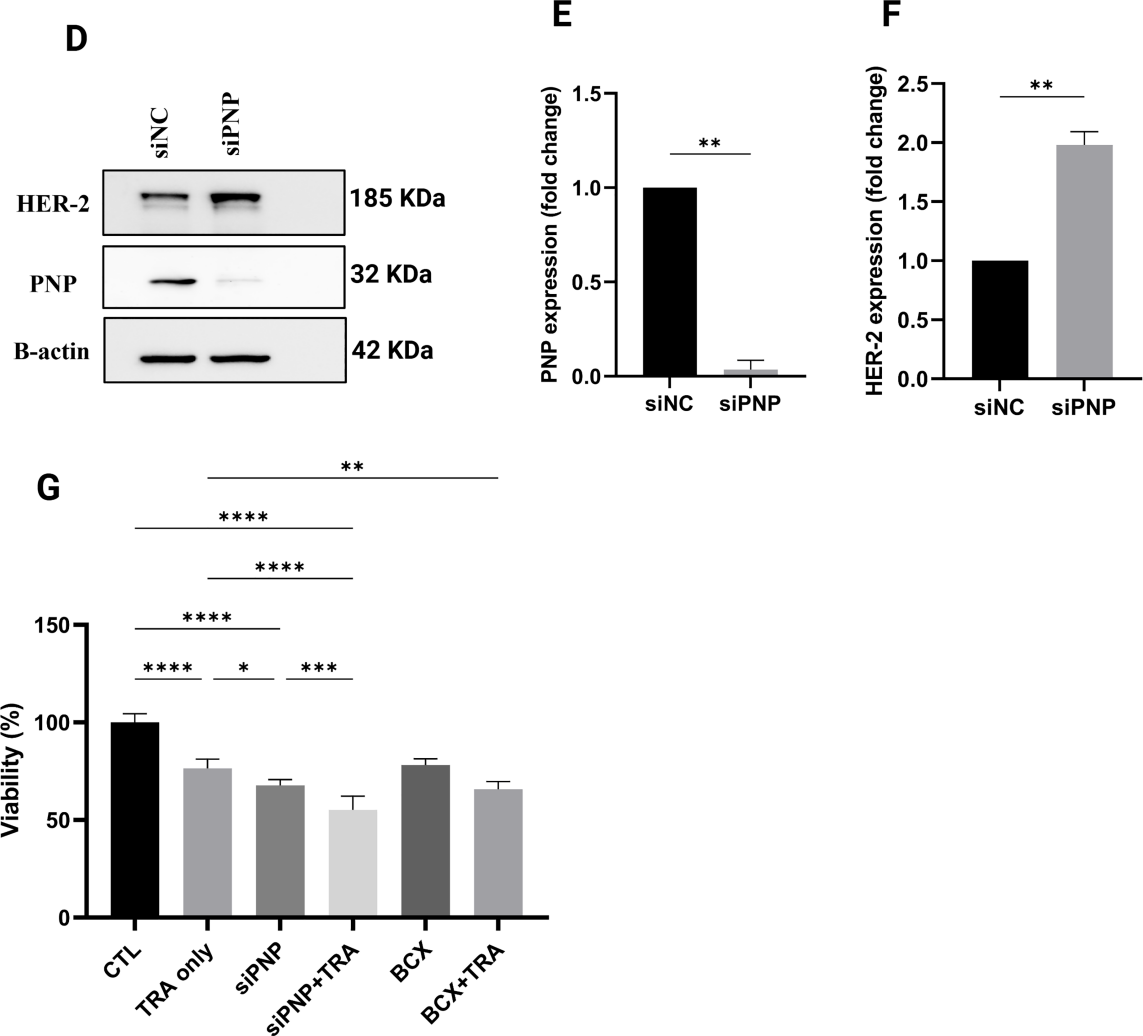
Correlation between PNP and HER-2 in SKBR3 cells. (**A**) Western blot analysis showing protein expression levels of HER-2 and PNP in SKBR3 cells treated with vehicle control (CTL), BCX-1777 (a PNP inhibitor), or trastuzumab (a HER-2 inhibitor). Quantification of protein expression levels of (**B**) PNP and (**C**) HER-2 in SKBR3 cells treated as in (**A**). (**D**) Western blot showing HER-2 and PNP protein levels after siRNA-mediated knockdown of PNP (siPNP) or negative control (siNC). Quantification of (**E**) PNP and (**F**) HER-2 expression in siPNP versus siNC conditions. (**G**) SKBR3 cell viability was assessed using the MTT assay and expressed as a percentage relative to untreated control (CTL) cells. Data shown are representative blots from three independent biological replicates (*n* = 3). Quantification represents mean ± SD. The data were analyzed using Student’s *t*-test or one-way ANOVA. *p-*value < 0.05 was considered significant. * reveals that *p*-value < 0.05, ** reveals that *p*-value < 0.01, *** reveals that *p*-value < 0.001, **** reveals that *p*-value < 0.0001. Original blots are presented in Fig. A4

To investigate the regulatory relationship between HER-2 expression and PNP, HER-2 was inhibited using trastuzumab, which resulted in a significant (*p* < 0.01) reduction in PNP by 0.6-fold, assessed by western blotting (Fig. 7A,B). Conversely, inhibition of PNP using BCX-1777 or siRNA-mediated knockdown resulted in significant (*p* < 0.01) upregulation of HER-2 by ~1.45 and 2-fold, respectively (Fig. 7C,F). These results suggest a reciprocal regulatory mechanism by which HER-2 promotes PNP expression, while PNP suppresses HER-2, indicating a negative feedback loop relevant to BC progression and therapeutic resistance.

### 3.8 Genetic and Pharmacologic Dual Inhibition Regimens of HER-2 and PNP Significantly Reduce Cell Viability in HER2-Positive BC Cells

To identify the effect of HER-2 and PNP inhibition on the viability of HER2-positive BC cells, SKBR3 cells were treated with trastuzumab (10 μg/mL), BCX-1777 (10 μM), or a combination of trastuzumab and BCX-1777. In parallel, PNP-silenced cells (siPNP) were assessed either treated with trastuzumab or not. Cell viability was assessed using the MTT assay (Fig. 7G).

Treatment with trastuzumab alone resulted in a significant (*p* < 0.0001) reduction in cell viability (23.6%) compared to the untreated cells (CTL). Silencing of *PNP* (siPNP) resulted in a greater decrease in viability (~30%). Notably, treatment of PNP-silenced cells with trastuzumab produced a pronounced and highly significant cytotoxic effect, reducing cell viability by ~45% compared to CTL (*p* < 0.0001), and significantly more than either treatment alone.

Treatment with BCX-1777 alone, a pharmacological PNP inhibitor, significantly reduces the cell viability by ~30%. A combination of BCX-1777 with trastuzumab resulted in further reduction in the cell viability (~34%, *p* < 0.01). These results demonstrate that inhibition of *PNP* enhances the cytotoxic effect of HER-2 inhibition.

## 4 Discussion

Recently, we have demonstrated the importance of PNP and its metabolic product, hypoxanthine, on EMT, migration, and invasion of MCF-7 cells [8]. Higher levels of hypoxanthine were identified in MDA-MB-231 (high metastatic cells) compared to MCF-7 (low metastatic cells). Herein, we investigate the relationship between PNP, a purine salvage pathway enzyme responsible for the biosynthesis of hypoxanthine [28], and BC aggressiveness and thereby invasion and metastasis using an *in silico* approach followed by experimental validation in BC tissues and cell lines.

Our *in silico* analysis showed a significant correlation between PNP and EMT markers. Cells exhibiting this phenotype enable them to migrate in collective clusters [29]. When these clusters enter the bloodstream, they can form groups of circulating tumor cells (CTCs). Cells with EMT phenotype are more aggressive and exhibit greater resistance to chemotherapeutic agents [29]. Genome-wide gene expression analysis and luciferase reporter assay identified PNP as a direct target of microRNA-1 and microRNA-133a and functions as an oncogene [16]. Knockdown of the PNP gene inhibited migration and invasion of prostate cancer and bladder cancer cell lines [16,30]. In line with this, we have also reported that silencing of PNP in MDA-MB-231 BC cell line is associated with a significant decrease in the protein expression of E-cadherin, N-cadherin, and vimentin [8]. These findings revealed the potential role of PNP in EMT.

It has been reported that enzymes involved in *de novo* nucleotide synthesis can promote BC stemness and metastasis [31]. However, the role of salvage nucleotide synthesis pathway enzymes in BC metastasis is minimally investigated. The mRNA level of *HPRT1*, a salvage pathway enzyme, was reported to be higher in BC patients compared to normal individuals, and the highest in TNBC compared to other BC subtypes [32]. It has been demonstrated that cancer patients exhibit significantly higher plasma PNP levels compared to normal individuals; however, no significant differences between cancer types were observed [12]. In line with this, our *in silico* analysis of normal and BC tissues indicated a significant increase in mRNA expression of *PNP* in BC tissues. Lymphocytes of patients with lung cancer and carcinoma of the larynx showed an elevated level of PNP [33]. In addition, PNP activity was found to be high in the lymphocytes of patients with bronchogenic carcinoma and non-Hodgkin lymphomas [14,15]. Moreover, PNP was found to be higher in the pancreatic juice from patients with pancreatic carcinoma compared to healthy controls [34]. In cancer patients, elevated plasma PNP levels may result from expression by cancer cells, lymphocytes, and macrophages [12].

Here, *in silico* analysis of transcriptomics data of 2509 patients with invasive breast carcinoma is consistent with our immunostaining of tissue samples from 103 BC patients. HER-2 positive-ER negative tumors, such as luminal B HER-2 positive and HER-2 overexpression, express higher levels of PNP than HER-2 negative-ER positive tumors, including luminal A and luminal B HER-2 negative, indicating a positive correlation between PNP and HER-2 and a negative correlation between PNP and ER. HER-2-overexpressing tumors are more aggressive than luminal subtypes and are associated with poor prognosis. TNBC is the most aggressive BC subtype [35]. Moreover, we have found that PNP is negatively correlated with ER and PR, while HER-2 is positively correlated with PNP. These results suggest that PNP is associated with BC aggressiveness.

Our study indicates a reciprocal regulatory relationship between HER-2 and PNP in BC. Particularly, PNP protein expression was significantly downregulated when HER-2 was inhibited with trastuzumab, indicating that HER-2 may positively regulate PNP expression either transcriptionally or post-translationally. HER-2 was significantly upregulated in response to both PNP gene silencing and chemical inhibition of PNP using BCX-1777, suggesting a possible compensatory or feedback mechanism. These results revealed that PNP might suppress HER-2 expression, possibly by interacting with oncogenic signaling through metabolic pathways.

This mutual relationship suggests a new regulatory pathway linking epidermal growth factor receptor signaling and purine metabolism in BC. When the nucleotide salvage pathway is impaired, the upregulation of HER-2 upon PNP inhibition might be a cellular attempt to preserve proliferative signaling. It is well noted that this type of interaction between metabolic enzymes and receptor tyrosine kinases is a characteristic of adaptive resistance in tumors [36]. The clinical relevance of this interaction is further supported by our immunohistochemical analysis of BC tissues, which reveals variable PNP expression among patients with HER-2-positive profiles. These results indicate that focusing on one HER-2/PNP axis node may have an unintended effect on other proteins, potentially affecting the effectiveness of treatment. Genetic silencing and pharmacological inhibition of PNP markedly reduced viability and synergized with trastuzumab, highlighting the importance of efficient PNP targeting. Therefore, combinatorial approaches that take into consideration the reciprocal regulation of HER-2 and PNP may enhance treatment efficacy and reduce resistance in HER-2-positive BC.

*In silico* results also demonstrated that PNP expression is high in patients with higher grades, confirming the association between PNP and BC aggressiveness. Metastatic BC is considered the last stage of BC (stage IV) [27]. With the advancement of BC stages, PNP expression was observed to be elevated, confirming its correlation with the BC’s invasiveness and metastatic potential. It was previously reported that PNP expression increased with the advancement of colon cancer stages [37]. In addition, *in silico* results demonstrated that PNP expression is positively associated with high proliferation of BC cells, indicated by the increase in the mitotic index Ki-67. Silencing of PNP was reported to inhibit the proliferation of prostate cancer cells [16]. Similarly, knockdown of PNP in bladder cancer cells resulted in a reduction of cell viability and apoptosis [30]. PNP inhibitors, including forodesine (BCX-1777), effectively inhibit cell growth by triggering apoptosis in patients with chronic lymphocytic leukemia [38]. Recently, phase I and II clinical trials have been conducted to evaluate the efficacy of these PNP inhibitors in treating patients with leukemia [39]. Observations from *p*atients with PNP deficiency, combined with both clinical and preclinical data on PNP inhibitors, support their application as agents in immuno-oncology and as adjuncts in vaccine usage [38].

The protein expression in different BC cell lines, representing various molecular subtypes of BC, was investigated. Compared to normal cells (HME1), BC cell lines exhibited elevated expression of PNP. Among these, the SKBR3 cell line showed the greatest increase, followed by MDA-MB-231, BT474, and MCF-7. The unique decrease in PNP expression in luminal A (MCF-7) suggests that MCF-7 cells have lower purine metabolism than other cell types. The observed differences might be partially attributed to the heterogeneity in hormonal receptor and HER-2 expressions among the different BC subtypes [40]. TNBC cells (MDA-MB-231) were reported to have a higher rate of purine metabolism compared to normal breast cells [41].

One major limitation of this study is that, despite integrating *in silico* analyses, immunohistochemistry, and *in vitro* validation, *in vivo* studies were not conducted to confirm the functional role of PNP–HER-2 interactions in BC progression and metastasis. Second, the mechanistic basis of the reciprocal regulation between PNP and HER-2 was not fully elucidated, and further exploration of the underlying signaling pathways is warranted. Third, the immunohistochemical validation was based on 103 tissue samples, which may not fully capture the molecular heterogeneity of BC across diverse patient populations, and the majority of the patients were of luminal subtypes.

## 5 Conclusions

This study identifies PNP as a predictive and prognostic biomarker in BC, with a strong association in disease aggressiveness, stages, and metastatic potential. The dual regulatory relationship between PNP and HER-2 suggests a previously unrecognized metabolic-oncogenic feedback loop. These findings underscore the potential of PNP as a therapeutic target, especially in HER-2-positive and triple-negative BCs. Future therapeutic strategies that co-target PNP and HER-2 may enhance treatment precision and reduce resistance, advancing personalized cancer care.

## Figures and Tables

**Figure A3:**
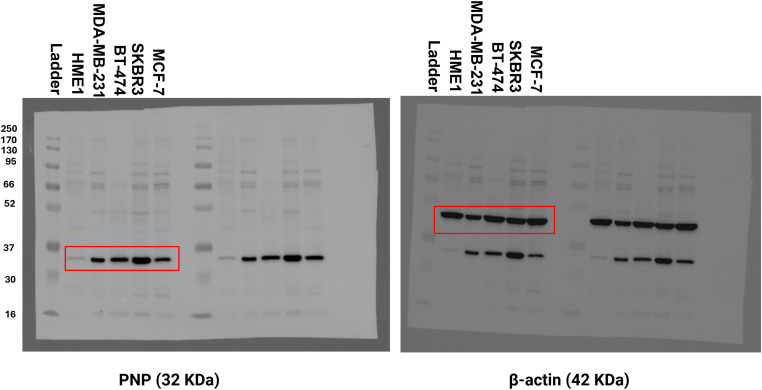
Original uncropped Western blot of Fig. 6. The figure shows the uncropped western blot of PNP and β-actin protein bands in different BC cell lines and normal cells

**Figure A4:**
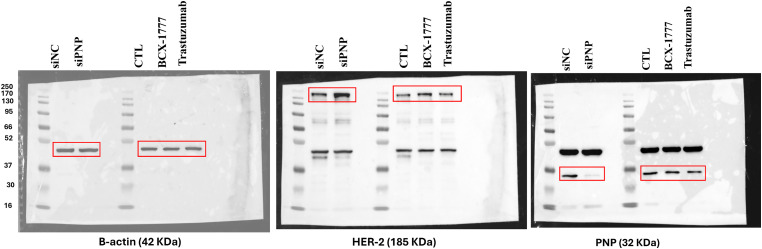
Original uncropped Western blot of Fig. 7. The figure shows the uncropped western blot of β-actin, HER-2, and β-actin protein bands in SKBR3 cells upon Knockdown of PNP and when the cells are treated with treatment with BCX-1777 or Trastuzumab

## Data Availability

Not applicable.
